# *catena*-Poly[[bis­(nitrato-κ*O*)cobalt(II)]-bis­[μ-1,4-bis­(pyridin-3-ylmeth­oxy)benzene-κ^2^ *N*:*N*′]]

**DOI:** 10.1107/S1600536811018630

**Published:** 2011-05-25

**Authors:** Ying Liu, Hong-Sen Zhang, Guang-Feng Hou, Jin-Sheng Gao

**Affiliations:** a Department of Materials and Chemistry Engineering, Heilongjiang Institute of Technology, Harbin 150050, People’s Republic of China; b Modern Analysis, Test and Research Center, Heilongjiang Institute of Science and Technology, Harbin 150027, People’s Republic of China; c College of Chemistry and Materials Science, Heilongjiang University, Harbin 150080, People’s Republic of China

## Abstract

In the title compound, [Co(NO_3_)_2_(C_18_H_16_N_2_O_2_)_2_]_*n*_, the Co^II^ ion is located on an inversion center and is six-coordinated in an octa­hedral environment defined by four N atoms of the pyridine rings and two O atoms of the nitrate anions. The ligands link the Co^II^ ions into a linear chain running along [201]. One O atom of the nitrate ligand is disordered over two positions with site-occupancy factors of 0.59 (4) and 0.41 (4).

## Related literature

For the synthesis and background to our study of flexible pyridyl-based aromatic ligands, see: Liu *et al.* (2010*a* ▶,*b* ▶); Yu *et al.* (2010 ▶). For the isotypic Cu(II) compound, see: Zou *et al.* (2011 ▶).
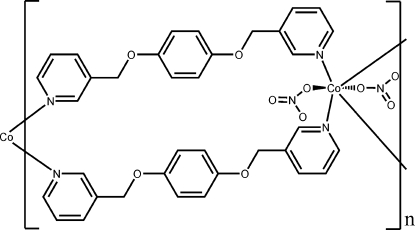


## Experimental

### 

#### Crystal data

[Co(NO_3_)_2_(C_18_H_16_N_2_O_2_)_2_]
*M* *_r_* = 767.61
Monoclinic, 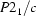

*a* = 8.3864 (17) Å
*b* = 16.751 (3) Å
*c* = 13.273 (5) Å
β = 115.26 (2)°
*V* = 1686.3 (8) Å^3^
*Z* = 2
Mo *K*α radiation
μ = 0.58 mm^−1^
*T* = 291 K
0.21 × 0.19 × 0.17 mm

#### Data collection

Rigaku R-AXIS RAPID diffractometer
Absorption correction: multi-scan (*ABSCOR*; Higashi, 1995 ▶) *T*_min_ = 0.888, *T*_max_ = 0.907
15667 measured reflections
3770 independent reflections
3176 reflections with *I* > 2σ(*I*)
*R*_int_ = 0.029

#### Refinement

*R*[*F* ^2^ > 2σ(*F* ^2^)] = 0.035
*wR*(*F* ^2^) = 0.093
*S* = 1.07
3770 reflections
251 parameters
12 restraints
H-atom parameters constrained
Δρ_max_ = 0.40 e Å^−3^
Δρ_min_ = −0.24 e Å^−3^

### 

Data collection: *RAPID-AUTO* (Rigaku, 1998 ▶); cell refinement: *RAPID-AUTO*; data reduction: *CrystalStructure* (Rigaku/MSC, 2002 ▶); program(s) used to solve structure: *SHELXS97* (Sheldrick, 2008 ▶); program(s) used to refine structure: *SHELXL97* (Sheldrick, 2008 ▶); molecular graphics: *SHELXTL* (Sheldrick, 2008 ▶); software used to prepare material for publication: *SHELXL97*.

## Supplementary Material

Crystal structure: contains datablocks I, global. DOI: 10.1107/S1600536811018630/ng5160sup1.cif

Structure factors: contains datablocks I. DOI: 10.1107/S1600536811018630/ng5160Isup2.hkl

Additional supplementary materials:  crystallographic information; 3D view; checkCIF report

## Figures and Tables

**Table 1:** Selected bond lengths (Å)

Co1—N2^i^	2.1307 (15)
Co1—O3	2.1682 (13)
Co1—N1	2.2016 (15)

Symmetry code: (i) 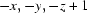.

**Fig. 1.**
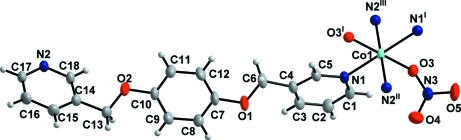
The molecular structure of the title compound, showing displacement ellipsoids at the 50% probability level for non-H atoms, disordered O5' atom has been omitted for clarity, Symmetry codes: (I) -2 - x, -y, -z; (II) -2 + x, y, -1 + z; (III) -x, -y, 1 - z.

**Fig. 2.**
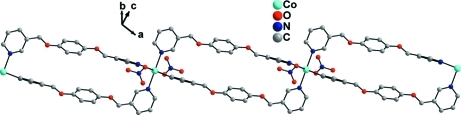
A partial packing view, showing the ribbon structure along [2 0 1] direction. Disordered O5' atoms and no involving H atoms have been omitted for clarity.

## supplementary crystallographic information

### Comment

The bridging compounds with rigid and flexible pyridyl-containing bidentate or multidentate organic spacers have assemble numerous interesting topology structures by coordination with metals and intermolecular supramolecular interaction. Our group focus attention on study of flexible pyridyl-based aromatic ligands, and obtained some isolated molecule, chain, plane and three-dimensional network structures (Liu *et al.*, 2010*a*; Liu *et al.*, 2010*b*; Yu *et al.*, 2010). Herein, as a continuing work for pyridyl ligands, we report the synthesis and crystal structure of the title compound, which is a isomorphic compound of our previous report (Zou *et al.*, 2011).

An asymmetric unit of the title compound consists of a 1,4-bis(pyridin-3-ylmethoxy)benzene molecule, a nitrate anion and a Co^II^ cation (Figure 1). The Co^II^ cation lie on an inversion center and is six-coordinated in the octahedral geometry environment defined by four N atoms of the pyridine derivatives and two O atoms of the nitrate anions (Table 1).

In the crystal, ribbon structures along [2 0 1] direction are built up by N-heterocyclic ligands linking Co^II^ cations (Figure 2).

### Experimental

The 1,4-bis(pyridin-3-ylmethoxy)benzene ligand was synthesized as the reference method (Liu *et al.*, 2010*a*): A mixture of 1,4-dihydroxybenzene (1.1 g, 10 mmol), 3-chloromethylpyridine hydrochloride (3.28 g, 20 mmol) and NaOH (1.6 g, 40 mmol) in acetonitrile (50 ml) was refluxed under nitrogen with stirring for 24 h. After cooling to room temperature, the solution was filtered and the residue was washed with acetonitrile for several times. The mixed filtrate was droped into 300 ml water solution to get the powder crude product. A total of 2.51 g (yield 86%) pure product was obtained by recrystallizing from the mixed solution of 10 ml water and 10 ml me thanol. The title compound was synthesized by reaction of 1,4-bis(pyridin-3-ylmethoxy)benzene ligand (0.29 g, 1.0 mmol) and Co(NO_3_)_2_^.^6H_2_O (0.29 g, 1.0 mmol) in 5 ml water and 5 ml me thanol mixed solution, and filtered after stirring for about 1 h. The filtate allowed to stand for four days under the room temperature to obtain pink block-like crystals suitable for X-ray analysis.

### Refinement

O5 atom of nitrate was disordered over two positions with site occupancy factors of *ca* 0.41 and 0.59,and then, the two positions were restraint refined with commond 'Iosr 0.005 O5 O4'. Four anormal reflection datas, namely, (7 0 4), (-7 5 3), (5 4 5), (7 5 2), have been omited. H atoms bound to C atoms were placed in calculated positions and treated as riding on their parent atoms, with C—H = 0.93 Å (aromatic); C—H = 0.97 Å (methylene), and with *U*_iso_(H) = 1.2*U*_eq_(C).

### Crystal data

**Table:**

[Co(NO_3_)_2_(C_18_H_16_N_2_O_2_)_2_]	*F*(000) = 794
*M**_r_* = 767.61	*D*_x_ = 1.512 Mg m^−^^3^
Monoclinic, *P*2_1_/*c*	Mo *K*α radiation, λ = 0.71073 Å
Hall symbol: -P 2ybc	Cell parameters from 13456 reflections
*a* = 8.3864 (17) Å	θ = 3.3–27.5°
*b* = 16.751 (3) Å	µ = 0.58 mm^−^^1^
*c* = 13.273 (5) Å	*T* = 291 K
β = 115.26 (2)°	Block, red
*V* = 1686.3 (8) Å^3^	0.21 × 0.19 × 0.17 mm
*Z* = 2	

### Data collection

**Table:**

Rigaku R-AXIS RAPID diffractometer	3770 independent reflections
Radiation source: fine-focus sealed tube	3176 reflections with *I* > 2σ(*I*)
graphite	*R*_int_ = 0.029
ω scans	θ_max_ = 27.5°, θ_min_ = 3.3°
Absorption correction: multi-scan (*ABSCOR*; Higashi, 1995)	*h* = −10→10
*T*_min_ = 0.888, *T*_max_ = 0.907	*k* = −21→21
15667 measured reflections	*l* = −16→17

### Refinement

**Table:**

Refinement on *F*^2^	Primary atom site location: structure-invariant direct methods
Least-squares matrix: full	Secondary atom site location: difference Fourier map
*R*[*F*^2^ > 2σ(*F*^2^)] = 0.035	Hydrogen site location: inferred from neighbouring sites
*wR*(*F*^2^) = 0.093	H-atom parameters constrained
*S* = 1.07	*w* = 1/[σ^2^(*F*_o_^2^) + (0.0468*P*)^2^ + 0.568*P*] where *P* = (*F*_o_^2^ + 2*F*_c_^2^)/3
3770 reflections	(Δ/σ)_max_ < 0.001
251 parameters	Δρ_max_ = 0.40 e Å^−^^3^
12 restraints	Δρ_min_ = −0.24 e Å^−^^3^

### Special details

**Table:**

Geometry. All e.s.d.'s (except the e.s.d. in the dihedral angle between two l.s. planes) are estimated using the full covariance matrix. The cell e.s.d.'s are taken into account individually in the estimation of e.s.d.'s in distances, angles and torsion angles; correlations between e.s.d.'s in cell parameters are only used when they are defined by crystal symmetry. An approximate (isotropic) treatment of cell e.s.d.'s is used for estimating e.s.d.'s involving l.s. planes.
Refinement. Refinement of *F*^2^ against ALL reflections. The weighted *R*-factor *wR* and goodness of fit *S* are based on *F*^2^, conventional *R*-factors *R* are based on *F*, with *F* set to zero for negative *F*^2^. The threshold expression of *F*^2^ > σ(*F*^2^) is used only for calculating *R*-factors(gt) *etc*. and is not relevant to the choice of reflections for refinement. *R*-factors based on *F*^2^ are statistically about twice as large as those based on *F*, and *R*- factors based on ALL data will be even larger.

### Fractional atomic coordinates and isotropic or equivalent isotropic displacement parameters (Å^2^)

**Table:**

	*x*	*y*	*z*	*U*_iso_*/*U*_eq_	Occ. (<1)
Co1	−1.0000	0.0000	0.0000	0.02593 (10)	
O1	−0.18789 (18)	−0.14784 (9)	0.32358 (15)	0.0595 (5)	
O2	0.52403 (19)	−0.11163 (9)	0.58551 (15)	0.0593 (5)	
O3	−1.20689 (16)	−0.06442 (7)	0.02231 (11)	0.0352 (3)	
O4	−1.2179 (2)	−0.18994 (9)	−0.01783 (15)	0.0581 (4)	
O5	−1.4545 (10)	−0.1223 (6)	−0.0743 (18)	0.063 (3)	0.41 (4)
N1	−0.78768 (18)	−0.07480 (9)	0.11956 (11)	0.0296 (3)	
N2	0.98946 (18)	−0.07047 (8)	0.86365 (11)	0.0281 (3)	
N3	−1.2934 (2)	−0.12828 (9)	−0.01457 (13)	0.0363 (3)	
C1	−0.8314 (2)	−0.13113 (11)	0.17496 (15)	0.0346 (4)	
H1	−0.9503	−0.1403	0.1556	0.041*	
C2	−0.7088 (2)	−0.17610 (12)	0.25912 (15)	0.0380 (4)	
H2	−0.7452	−0.2152	0.2945	0.046*	
C3	−0.5319 (2)	−0.16281 (11)	0.29067 (14)	0.0353 (4)	
H3	−0.4472	−0.1925	0.3476	0.042*	
C4	−0.4826 (2)	−0.10430 (11)	0.23586 (14)	0.0318 (4)	
C5	−0.6145 (2)	−0.06312 (10)	0.15006 (14)	0.0318 (4)	
H5	−0.5813	−0.0253	0.1114	0.038*	
C6	−0.2936 (2)	−0.08121 (12)	0.27107 (17)	0.0420 (5)	
H6A	−0.2746	−0.0656	0.2066	0.050*	
H6B	−0.2633	−0.0364	0.3222	0.050*	
C7	−0.0111 (2)	−0.13537 (11)	0.38925 (16)	0.0379 (4)	
C8	0.0822 (2)	−0.20193 (11)	0.44505 (16)	0.0358 (4)	
H8	0.0244	−0.2504	0.4378	0.043*	
C9	0.2614 (2)	−0.19664 (11)	0.51163 (15)	0.0353 (4)	
H9	0.3245	−0.2416	0.5485	0.042*	
C10	0.3463 (2)	−0.12396 (11)	0.52298 (15)	0.0372 (4)	
C11	0.2531 (3)	−0.05720 (12)	0.46827 (18)	0.0438 (5)	
H11	0.3105	−0.0085	0.4768	0.053*	
C12	0.0744 (3)	−0.06280 (12)	0.40079 (18)	0.0440 (5)	
H12	0.0117	−0.0180	0.3632	0.053*	
C13	0.6332 (2)	−0.17831 (11)	0.62877 (16)	0.0394 (4)	
H13A	0.6360	−0.2106	0.5689	0.047*	
H13B	0.5902	−0.2109	0.6724	0.047*	
C14	0.8133 (2)	−0.14676 (10)	0.70073 (14)	0.0314 (4)	
C15	0.9609 (2)	−0.16228 (11)	0.68233 (15)	0.0379 (4)	
H15	0.9519	−0.1924	0.6213	0.045*	
C16	1.1220 (2)	−0.13215 (11)	0.75657 (16)	0.0381 (4)	
H16	1.2228	−0.1416	0.7457	0.046*	
C17	1.1322 (2)	−0.08808 (10)	0.84652 (15)	0.0317 (4)	
H17	1.2418	−0.0698	0.8973	0.038*	
C18	0.8350 (2)	−0.09926 (10)	0.79096 (14)	0.0298 (3)	
H18	0.7352	−0.0866	0.8016	0.036*	
O5'	−1.4489 (10)	−0.1298 (4)	−0.0282 (16)	0.065 (2)	0.59 (4)

### Atomic displacement parameters (Å^2^)

**Table:**

	*U*^11^	*U*^22^	*U*^33^	*U*^12^	*U*^13^	*U*^23^
Co1	0.01535 (16)	0.03043 (17)	0.02660 (16)	0.00104 (12)	0.00375 (12)	−0.00271 (12)
O1	0.0175 (7)	0.0419 (8)	0.0931 (12)	0.0015 (6)	−0.0013 (7)	0.0135 (8)
O2	0.0236 (8)	0.0371 (8)	0.0823 (11)	−0.0020 (6)	−0.0107 (7)	0.0046 (7)
O3	0.0260 (7)	0.0340 (7)	0.0433 (7)	−0.0036 (5)	0.0126 (5)	−0.0017 (5)
O4	0.0532 (10)	0.0361 (8)	0.0798 (11)	0.0038 (7)	0.0233 (9)	−0.0053 (7)
O5	0.022 (2)	0.072 (3)	0.078 (5)	−0.0074 (18)	0.006 (3)	−0.001 (3)
N1	0.0200 (7)	0.0342 (7)	0.0296 (7)	0.0021 (5)	0.0057 (6)	−0.0005 (5)
N2	0.0196 (7)	0.0311 (7)	0.0289 (7)	0.0012 (5)	0.0057 (5)	−0.0019 (5)
N3	0.0251 (8)	0.0375 (8)	0.0442 (8)	−0.0004 (6)	0.0126 (7)	0.0024 (6)
C1	0.0203 (9)	0.0422 (10)	0.0376 (9)	−0.0013 (7)	0.0090 (7)	0.0001 (7)
C2	0.0311 (10)	0.0434 (10)	0.0373 (9)	−0.0023 (8)	0.0125 (8)	0.0068 (7)
C3	0.0258 (10)	0.0418 (10)	0.0304 (8)	0.0041 (7)	0.0045 (7)	0.0063 (7)
C4	0.0199 (9)	0.0374 (9)	0.0329 (8)	0.0024 (7)	0.0064 (7)	0.0012 (7)
C5	0.0216 (9)	0.0350 (9)	0.0348 (8)	0.0024 (7)	0.0082 (7)	0.0053 (7)
C6	0.0196 (9)	0.0469 (11)	0.0503 (11)	0.0034 (8)	0.0060 (8)	0.0123 (8)
C7	0.0164 (9)	0.0434 (10)	0.0462 (10)	0.0015 (7)	0.0059 (7)	0.0062 (8)
C8	0.0248 (9)	0.0341 (9)	0.0441 (10)	−0.0027 (7)	0.0106 (8)	0.0039 (7)
C9	0.0250 (9)	0.0352 (9)	0.0370 (9)	0.0036 (7)	0.0047 (7)	0.0057 (7)
C10	0.0186 (9)	0.0406 (10)	0.0397 (9)	0.0001 (7)	0.0001 (7)	−0.0005 (7)
C11	0.0259 (10)	0.0345 (10)	0.0584 (12)	−0.0030 (7)	0.0059 (9)	0.0041 (8)
C12	0.0250 (10)	0.0361 (10)	0.0585 (12)	0.0056 (8)	0.0058 (9)	0.0120 (8)
C13	0.0255 (10)	0.0377 (10)	0.0407 (10)	0.0017 (7)	0.0003 (8)	−0.0080 (7)
C14	0.0229 (9)	0.0315 (8)	0.0315 (8)	0.0022 (7)	0.0038 (7)	−0.0022 (6)
C15	0.0336 (11)	0.0393 (10)	0.0382 (9)	0.0037 (8)	0.0128 (8)	−0.0091 (7)
C16	0.0266 (10)	0.0388 (10)	0.0505 (11)	0.0028 (7)	0.0182 (8)	−0.0058 (8)
C17	0.0198 (8)	0.0313 (9)	0.0381 (9)	0.0004 (6)	0.0067 (7)	−0.0019 (7)
C18	0.0182 (8)	0.0366 (9)	0.0302 (8)	0.0008 (6)	0.0059 (6)	−0.0024 (6)
O5'	0.0261 (19)	0.076 (2)	0.093 (5)	−0.0091 (15)	0.025 (2)	−0.003 (3)

### Geometric parameters (Å, °)

**Table:**

Co1—N2^i^	2.1307 (15)	C4—C5	1.386 (2)
Co1—N2^ii^	2.1307 (15)	C4—C6	1.500 (3)
Co1—O3	2.1682 (13)	C5—H5	0.9300
Co1—O3^iii^	2.1682 (13)	C6—H6A	0.9700
Co1—N1	2.2016 (15)	C6—H6B	0.9700
Co1—N1^iii^	2.2016 (15)	C7—C8	1.382 (3)
O1—C7	1.378 (2)	C7—C12	1.386 (3)
O1—C6	1.411 (2)	C8—C9	1.384 (3)
O2—C10	1.377 (2)	C8—H8	0.9300
O2—C13	1.402 (2)	C9—C10	1.386 (3)
O3—N3	1.2679 (19)	C9—H9	0.9300
O4—N3	1.222 (2)	C10—C11	1.380 (3)
O5—O5'	0.606 (9)	C11—C12	1.383 (3)
O5—N3	1.245 (7)	C11—H11	0.9300
N1—C1	1.340 (2)	C12—H12	0.9300
N1—C5	1.346 (2)	C13—C14	1.497 (2)
N2—C18	1.332 (2)	C13—H13A	0.9700
N2—C17	1.343 (2)	C13—H13B	0.9700
N2—Co1^iv^	2.1307 (15)	C14—C18	1.384 (2)
N3—O5'	1.238 (5)	C14—C15	1.385 (3)
C1—C2	1.376 (3)	C15—C16	1.384 (3)
C1—H1	0.9300	C15—H15	0.9300
C2—C3	1.377 (3)	C16—C17	1.375 (3)
C2—H2	0.9300	C16—H16	0.9300
C3—C4	1.385 (3)	C17—H17	0.9300
C3—H3	0.9300	C18—H18	0.9300
			
N2^i^—Co1—N2^ii^	180.00 (7)	O1—C6—C4	107.92 (16)
N2^i^—Co1—O3	84.57 (5)	O1—C6—H6A	110.1
N2^ii^—Co1—O3	95.43 (5)	C4—C6—H6A	110.1
N2^i^—Co1—O3^iii^	95.43 (5)	O1—C6—H6B	110.1
N2^ii^—Co1—O3^iii^	84.57 (5)	C4—C6—H6B	110.1
O3—Co1—O3^iii^	180.00 (9)	H6A—C6—H6B	108.4
N2^i^—Co1—N1	88.60 (6)	O1—C7—C8	115.30 (17)
N2^ii^—Co1—N1	91.40 (6)	O1—C7—C12	124.69 (17)
O3—Co1—N1	93.81 (5)	C8—C7—C12	120.00 (17)
O3^iii^—Co1—N1	86.19 (5)	C7—C8—C9	120.22 (17)
N2^i^—Co1—N1^iii^	91.40 (6)	C7—C8—H8	119.9
N2^ii^—Co1—N1^iii^	88.60 (6)	C9—C8—H8	119.9
O3—Co1—N1^iii^	86.19 (5)	C8—C9—C10	119.56 (17)
O3^iii^—Co1—N1^iii^	93.81 (5)	C8—C9—H9	120.2
N1—Co1—N1^iii^	180.00 (13)	C10—C9—H9	120.2
C7—O1—C6	118.18 (15)	O2—C10—C11	114.82 (17)
C10—O2—C13	118.47 (15)	O2—C10—C9	124.82 (17)
N3—O3—Co1	136.62 (11)	C11—C10—C9	120.36 (17)
O5'—O5—N3	75.2 (11)	C10—C11—C12	119.99 (18)
C1—N1—C5	116.76 (15)	C10—C11—H11	120.0
C1—N1—Co1	118.02 (12)	C12—C11—H11	120.0
C5—N1—Co1	124.85 (11)	C11—C12—C7	119.86 (18)
C18—N2—C17	117.35 (15)	C11—C12—H12	120.1
C18—N2—Co1^iv^	119.38 (11)	C7—C12—H12	120.1
C17—N2—Co1^iv^	123.27 (11)	O2—C13—C14	106.54 (15)
O4—N3—O5'	120.5 (3)	O2—C13—H13A	110.4
O4—N3—O5	118.9 (5)	C14—C13—H13A	110.4
O5'—N3—O5	28.3 (4)	O2—C13—H13B	110.4
O4—N3—O3	120.46 (16)	C14—C13—H13B	110.4
O5'—N3—O3	117.9 (3)	H13A—C13—H13B	108.6
O5—N3—O3	117.7 (5)	C18—C14—C15	117.62 (16)
N1—C1—C2	123.13 (17)	C18—C14—C13	118.62 (16)
N1—C1—H1	118.4	C15—C14—C13	123.76 (16)
C2—C1—H1	118.4	C16—C15—C14	118.72 (16)
C1—C2—C3	119.57 (17)	C16—C15—H15	120.6
C1—C2—H2	120.2	C14—C15—H15	120.6
C3—C2—H2	120.2	C17—C16—C15	119.63 (17)
C2—C3—C4	118.62 (16)	C17—C16—H16	120.2
C2—C3—H3	120.7	C15—C16—H16	120.2
C4—C3—H3	120.7	N2—C17—C16	122.36 (16)
C3—C4—C5	118.15 (16)	N2—C17—H17	118.8
C3—C4—C6	122.12 (16)	C16—C17—H17	118.8
C5—C4—C6	119.62 (16)	N2—C18—C14	124.24 (16)
N1—C5—C4	123.73 (16)	N2—C18—H18	117.9
N1—C5—H5	118.1	C14—C18—H18	117.9
C4—C5—H5	118.1	O5—O5'—N3	76.5 (11)

Symmetry codes: (i) −*x*, −*y*, −*z*+1; (ii) *x*−2, *y*, *z*−1; (iii) −*x*−2, −*y*, −*z*; (iv) *x*+2, *y*, *z*+1.
